# Thunder-Fire Moxibustion for Cervical Spondylosis: A Systematic Review and Meta-Analysis

**DOI:** 10.1155/2020/5816717

**Published:** 2020-02-11

**Authors:** Ruina Huang, Yunxuan Huang, Ruijia Huang, Shaofen Huang, Xiaojun Wang, Xiaojiang Yu, Danghan Xu, Xinghua Chen

**Affiliations:** ^1^The Eighth Affiliated Hospital of Sun Yat-Sen University, Shenzhen, China; ^2^The First Affiliated Hospital of Guangzhou University of Chinese Medicine, Guangzhou, China; ^3^The First Affiliated Hospital of Shantou University Medical College, Shantou, China; ^4^Shenzhen Hospital of Guangzhou University of Chinese Medicine, Shenzhen, China

## Abstract

**Background:**

Cervical spondylosis (CS) refers to the degenerative changes in the cervical spinal column, which affect the majority of middle-aged and elderly people. Thunder-fire moxibustion originated from thunder-fire miraculous needle, which has been applied widely for treating pain syndromes for thousands of years. The aim of our research is to provide evidence to assess the efficacy and safety of thunder-fire moxibustion in treating CS. *Methods and analysis.* Retrieved literature databases included Cochrane Library, MEDLINE, Web of Science, EBSCO, EBASE, Springer, PubMed, WFDP, CNKI, VIP, and CBM. The period of retrieval was from the establishment of the database to December 2018. Randomized controlled trials which compared thunder-fire moxibustion and other therapies in CS were included. The quality of inclusive trials was accessed though a Cochrane risk of bias tool. According to the test results of heterogeneity, a random effect model or fixed effect model was used to analyze the data.

**Results:**

Meta-analysis was conducted for the total effective rate of thunder-fire moxibustion, traditional Chinese medicine syndrome score, pain score, satisfaction score, and score of the symptoms and functional rehabilitation of cervical vertigo. The analysis results were as follows: compared with other therapies, the efficacy of thunder-fire moxibustion was statistically significant, total effective rate increased (OR = 2.48; 95% CI [1.80, 3.41]; *P* < 0.00001), traditional Chinese medicine syndrome score decreased (SMD = −3.05; 95% CI[−4.18, −1.93]; *P* < 0.00001), traditional Chinese medicine syndrome score decreased (SMD = −3.05; 95% CI[−4.18, −1.93]; *P* < 0.00001), traditional Chinese medicine syndrome score decreased (SMD = −3.05; 95% CI[−4.18, −1.93]; *P* < 0.00001), traditional Chinese medicine syndrome score decreased (SMD = −3.05; 95% CI[−4.18, −1.93]; *P* < 0.00001), traditional Chinese medicine syndrome score decreased (SMD = −3.05; 95% CI[−4.18, −1.93];

**Conclusion:**

Based on the existing evidence, the curative effect and safety of thunder-fire moxibustion on CS were statistically significant. We should interpret the results scrupulously because of the low evidence level. Large-scale, high-quality, rigorous RCTs with long-term follow-up should be performed in the future.

## 1. Introduction

Cervical spondylosis (CS) is a chronic cervical spine disc degenerative pathology, which influences the vertebral bodies and cervical intervertebral discs. It may develop into bone spur production, protrusion of intervertebral disc, and spinal cord compression [1]. The most prevalent symptoms are headaches, neck stiffness, numbness, neck and/or arm pain, neck and/or arm tingling, and bladder function problems [2]. CS is a common disease in middle-aged and senile populations. About 50% of people over 40 suffer from cervical spondylosis and about 85% of people over 60 were diagnosed with cervical spondylosis by radiology [3]. Age plays a double role in cervical spondylosis development. The morbidity of cervical spondylosis grows with age before 50 years old, while it does the opposite after 50 [4]. As the spine exposed to increasing loads with aging, it may lead to hypertrophy of the facet joints, ligamentum flavum, and posterior longitudinal ligament. The diameter of the cervical canal decreases, and then spinal cord compression develops [5]. The etiological factors include age, occupation, behavior, heredity, and sports-related activities [6–8]. Many asymptomatic CS patients may be diagnosed lingeringly because it attacks insidiously. So, sufferers usually have serious symptoms and permanent disability when they seek medical treatment [9]. Treatment for CS abroad includes conservative and surgical therapy. Conservative therapy consists of rest, immobilization of the cervical spine, drug therapy, and physical therapy [10]. Surgery can cure the core symptoms of CS, but there are still marginal symptoms, such as Barre-Lieou syndrome, tinnitus, blurred vision, cervical angina, cervical migraine, etc., [11]. Surgery is expensive and not appropriate for every patient [12]. Although these conservative therapies have demonstrated some total effective rates, these treatments are not always effective and even have some side effects.

Moxibustion is an ancient Chinese medicine therapy with a history of more than four thousand years. Moxibustion is to place the moxa cones on some acupoints of the body, and then ignite the moxa cones. Generally, the moxa cone is removed before it burns out, and it is replaced with a new moxa cone. There is another method of moxibustion, where the therapist holds a cigar-like moxa stick and applies moxibustion at the acupoints. Moxibustion points are usually selected according to the diseases to be treated. In traditional Chinese medicine, meridians and collaterals are channels to move qi and blood and connect viscera, body surface, and all parts of the body and are regulating systems of human functions. Moxibustion can promote the dredging of meridians and collaterals, improve local microcirculation, and accelerate the absorption of inflammatory media, so as to reduce local inflammatory response and relieve pain. In addition, moxibustion also has the effect of infrared resonance radiation. During the treatment process, it can promote the resonance of abnormal functional cells, make them undergo apoptosis, and at the same time, it can promote the activation and regeneration of tissue cells, so as to accelerate the local tissue metabolism and promote the functional recovery.

Thunder-fire moxibustion originated from the thunder-fire miraculous needle which had been used for thousands of years. It is a new moxibustion therapy ameliorated by Profession Zhao Shibi due to her experience of decades of medical practice [13]. Thunder-fire moxibustion is widely used in China to treat diseases, such as otolaryngological diseases, osteoarthritis, and gynecological diseases, and pain caused by any disease [14–19]. Different from the traditional moxibustion stick, the thunder-fire moxibustion adds agallochum, eaglewood, banksia rose, dried ginger, virgate wormwood herb, and stem of capillary sagebrush to a moxa stick [20]. These drugs play a role in warming meridians and dredging collaterals. When burning, thunder-fire moxibustion can produce strong firepower, thermal infrared radiation force, and medicine chemical factor [21]. The heat of thunder-fire moxibustion can improve blood circulation and stimulate related acupoints to activate meridians. The drug of thunder-fire moxibustion, therefore, penetrates and reaches the acupoints with a high drug concentration area in the human body surface. According to the theory of traditional Chinese medicine, the comprehensive effect of these factors can act on meridians and acupoints to raise local temperature, stimulate local blood circulation and lymph circulation, and accelerate metabolism so as to achieve the function of warming meridians to dredge collaterals, dispel coldness, eliminate wind-dampness, activate yang, transform qi, and adjust physical enginery [22, 23]. At the same time, special treatment techniques, such as bird pecking, rotation, and array, are used to mend the curative effect.

Previous studies had shown the effect of thunder-fire moxibustion in CS. However, the effectiveness of thunder-fire moxibustion in treating CS is still contentious because of inadequate sample size, low methodological quality, and lack of high-quality study in related clinical trials. As far as we know, there is no systematic review of thunder-fire moxibustion for CS. We, therefore, aim to evaluate the safety and effectiveness of thunder-fire moxibustion on CS and help clinicians to make recommendations and promote the research progress in thunder-fire moxibustion.

## 2. Methods

### 2.1. Study Registration

This systematic review and meta-analysis were registered at the International Prospective Register of Systematic Reviews (CRD42018115686). The report is composited based on the Preferred Reporting Items for Systematic Reviews and Meta-Analyses (PRISMA) guide [24].

### 2.2. Eligibility Criteria

#### 2.2.1. Types of Study

All randomized controlled trials (RCTs) that examine thunder-fire moxibustion efficacy in the treatment of CS were included.

#### 2.2.2. Types of Participants

Researches on adults, who had CS, were included. It had no limitation of gender, race, and type of cervical spondylosis. Trials containing patients with pregnancy, cancer, kidney disease, or liver disease were excluded.

#### 2.2.3. Types of Interventions

The intervention group adopts single thunder-fire moxibustion or thunder-fire moxibustion combined with other therapies, while the control group receives other therapies except thunder-fire moxibustion, such as usual care, acupuncture, moxibustion, medication or physical therapy, and so on.

#### 2.2.4. Types of Outcome Measures

We analyzed the total effective rate of the thunder-fire moxibustion, traditional Chinese medicine syndrome score [25], pain score, satisfaction score, and score of the symptoms and functional rehabilitation of cervical vertigo. Three outcome measures were positive indicators, such as total effective rate, satisfaction score, and score of the symptoms and functional rehabilitation of cervical vertigo, and the other two were negative indicators, such as pain score and traditional Chinese medicine syndrome score. Among the positive indicators, the higher the score, the better the effect of the intervention. Among the negative indicators, the lower the score, the better the effect of the intervention. The definitions of “total effective rate” in each study were different, including improvement of symptoms and signs such as vertigo, headache, neck pain, the neck and shoulder movement function, and ability to work normally. Although the definition of “total efficiency” was not the same in each study, they all referred to the remission of symptoms, the recovery of daily work and life. The traditional Chinese medicine syndrome score was measured by the TCM syndrome score and Neck Pain Questionnaire. The pain scores were assessed by the Numeric Rating Scale (NRS) [26] or Visual Analog Scale (VAS) [27]. The satisfaction score was evaluated by the hospital self-made satisfaction questionnaire. The score of the symptoms and functional rehabilitation of cervical vertigo were the evaluated by cervical vertigo symptom and function assessment scale.

### 2.3. Data Sources

The literature search was conducted from inception to December 2018 in the corresponding databases including Cochrane Library, MEDLINE, Web of Science, EBSCO, EBASE, Springer, PubMed, Wanfang Digital Periodicals Database (WFDP), China National Knowledge Infrastructure Database (CNKI), Chinese Biological Medicine Database (CBM), and VIP Database. The projects of RCTs were also selected in the languages of English or Chinese.

### 2.4. Search Strategy

All researchers worked together to develop retrieval terms and strategies based on the Cochrane Manual. We used the following search terms: Cervical Spondylosis (e.g., Cervical Spondylosis, Spondylosis, Cervical, Spondylosis Deformans) and Thunder-fire Moxibustion (e.g., Thunder-fire Moxibustion, Leihou Moxibustion). We used the search terms with the same meaning to retrieve domestic databases.  Table 1 shows us the search strategy of PubMed.

### 2.5. Data Collection and Analysis

#### 2.5.1. Research Selection

Before searching studies, investigators had discussed and decided screening criteria in the group. Firstly, the retrieved literatures were imported into the literature management system of EndNote X7 in order to delete duplicate documents. Secondly, two of the researchers (HR and HY) read the titles and abstracts of included literatures to exclude unqualified studies. Thirdly, these two researchers obtained and read the full text, and discussed in the group to select qualified studies. One researcher (CX) contacted the author to obtain relevant information of the researches as there was lack of related data. Any branching between researchers was solved by discussion or consulting a senior reviewer (CX).

#### 2.5.2. Data Extraction

We had made a data collection form to record the data of selected studies such as the first author, published year, sample size, age, intervention characteristics of both groups, moxibustion method, duration of treatment outcome measures, and adverse events, before extracting the useful information. The data extraction was completed by two reviewers (HR and HY) independently, and the extracted information was rechecked after finished. The divergence of opinion was solved through consulting the senior researcher (CX). If related data were deficient, one reviewer (CX) got in touch with the writers of articles for lost information through telephone or email. If we could not obtain sufficient information after hard work, our researchers processed the existing information and then explained the result.

#### 2.5.3. The Assessment for Risk of Bias

The Cochrane's risk of bias (ROB) was used as a tool to evaluate the risk of deviation [28]. Two of the researchers (HR and HY) evaluated the methodological quality of the including literatures on their own. We evaluated the following projects: random sequence generation, allocation concealment, blinding of researchers, subject and outcome assessment, incomplete outcome information, selective result reporting, and other sources of bias.

#### 2.5.4. Data Analysis and Synthesis

The collected data were analyzed though Review Manager (Version 5.3). Based on the result, the total effective rate, traditional Chinese medicine syndrome score, pain score, satisfaction score, and score of the symptoms and functional rehabilitation of cervical vertigo were analyzed, respectively. We used odds ratio (OR) to deal with dichotomous variable and mean differences (MDs) to deal with continuous variable. At the same time, 95% CI was calculated. We used the forest plot to explain treatment effect. When there was potential heterogeneity (clinical and methodological), due to the weak power of the Q statistics to detect heterogeneity, we should avoid using *I*^2^ to determine the model selection. If the number of studies involved was small, it may not be able to accurately estimate the variance between studies, and the fixed effect model is the first choice. *P* < 0.05 showed that there was a statistically significant difference. When substantial heterogeneity exited, we conducted subgroup analysis or sensitivity analysis to explore heterogeneity sources. We performed subgroup analysis according to the duration of treatment. If we could not find some resources, we reported a narrative description of the included researches, instead of conducting subgroup analysis. A sensitivity analysis through the leave-one-out method was carried out while substantive heterogeneity existed among trials. When there were more than ten included researches, we used the funnel plot to illustrate the reporting biases. The symmetry of the funnel plot reflected reporting bias. If the funnel plot was symmetrical, there was no bias, and vice versa.

### 2.6. Level of Evidence

We assessed the evidence level with the help of Grading of Recommendations, Assessment, Development, and Evaluation (GRADE) [29]. The level of evidence from low to high was classified into 4 grades: very low, low, moderate, and high. Evidence level was assessed based on eight factors. Risk of bias, imprecision, indirectness, inconsistency, and publication bias were downgrading factors. The remaining three were upgrading factors, which were dose-response gradient, large effect, and plausible confounding, which would change the effect.

## 3. Results

### 3.1. Search Results

**Figure 1:**
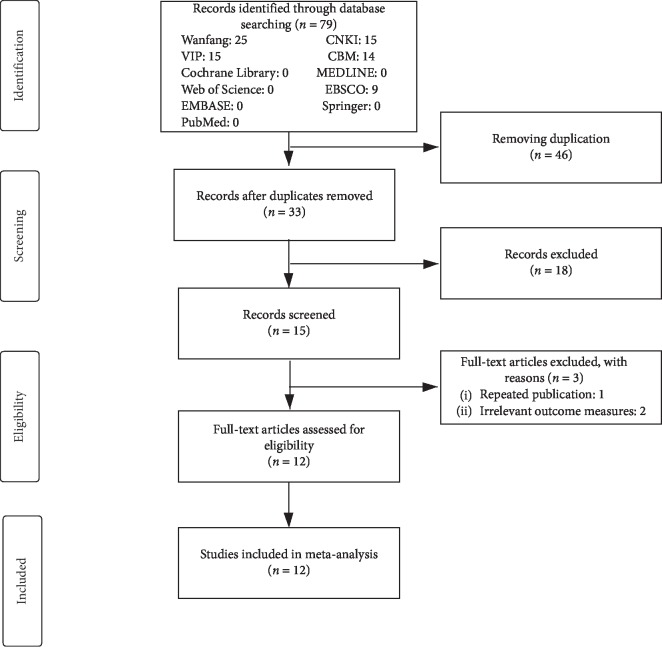
Flow diagram of studies identified.

Seventy-nine articles were retrieved in the database, 15 from CNKI, 15 from VIP, 25 from WFDP, 14 from CBM, 1 from Cochrane Library, 9 from EBSCO, 0 from MEDLINE, 0 from Web of Science, 0 from EBASE, 0 from Springer, and 0 from PubMed. Forty-six articles were deleted due to duplication. After reading the titles and abstracts, 18 irrelevant trials were excluded. Fifteen articles remained were read in full. After further reading, 1 article was excluded for repeated publication and 2 for the inappropriateness of outcome indicators. There were 12 articles including meta-analysis at last (Figure 1) [30–41].

### 3.2. Study Characteristics

The table shows the characteristics of the researches. All of the trials included had been conducted in China and published from 2003 to 2018. A total of 12 articles included 1304 patients. Seven trials [30–33, 36, 39, 41] had a treatment duration less than or equal to 2 weeks, and 5 trials [34, 35, 37, 38, 40] had a treatment duration more than 2 weeks. The baseline data between the two groups in every research were comparable.

Ten researches [30–33, 35–40] measured total effective rate, 4 researches [31, 32, 36, 38] measured traditional Chinese medicine syndrome score, 4 researches [30, 31, 38, 40] measured pain score, 2 researches [32, 33] measured satisfaction score, and 2 researches [34, 41] measured the score of the symptoms and functional rehabilitation of cervical vertigo.  Table 2 provides a detailed summary of the inclusive researches.

### 3.3. Risk of Bias Assessments

**Figure 2:**
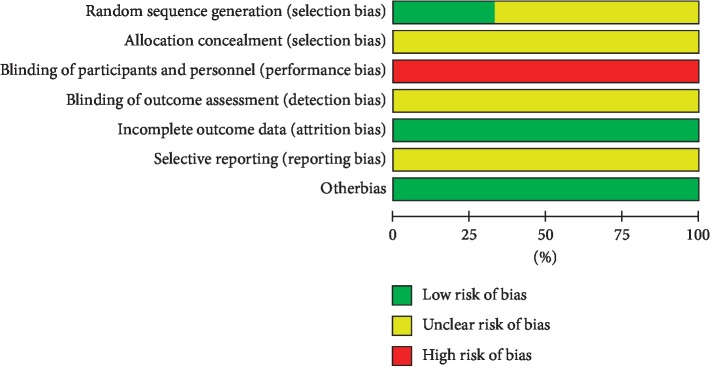
Risk of bias of the included studies.

**Figure 3:**
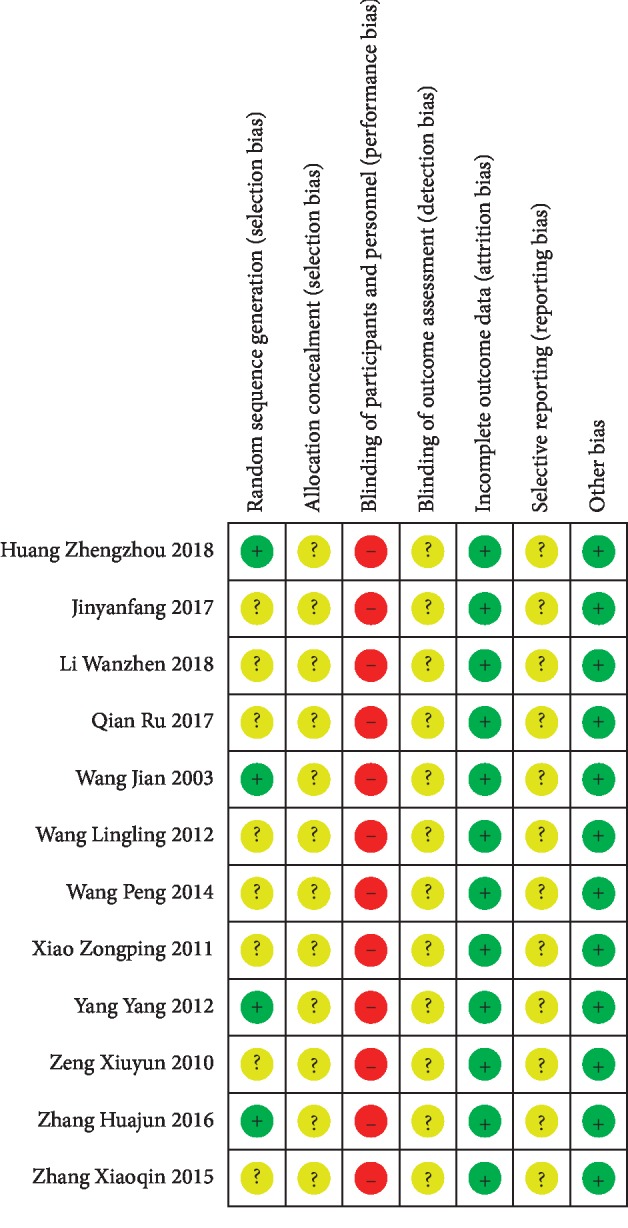
Risk of bias summary.

Four trials [30, 34, 38, 40] using the random number table were identified as low risk of bias and the remaining 8 trials [31–33, 35–37, 39, 41] were identified as unclear risk of bias. All trials did not indicate allocation concealment. Due to the particularity of intervention, all trials were unable to implement blinding, so the blinding of the researchers and subjects was assessed as high risk. The data in all studies were inadequate to assess the blindness of the outcome assessors, so we rated it as high risk. We considered all researches to be of low attrition bias. Since there was insufficient information to determine the selective reporting, we assessed all researches at unclear risk of bias. We thought that all trials have a low bias risk in other sources of bias (Figures 2 and 3).

## 4. Outcomes

### 4.1. Total Effective Rate

**Figure 4:**
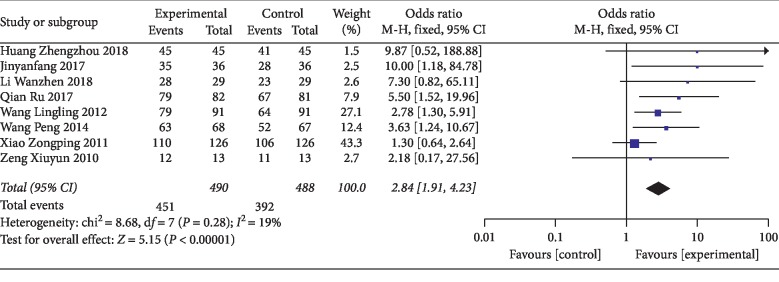
Forest plots of total effective rate (less than or equal to two weeks).

**Figure 5:**
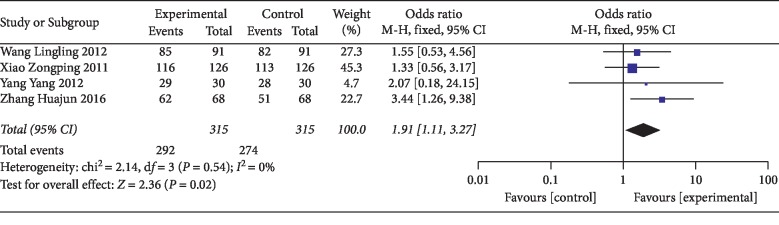
Forest plots of total effective rate (more than two weeks).

The total effective rate was reported in 10 [30–33, 35–40] out of 12 studies. Two of these studies [35, 37] reported the total effective rate for both two and three weeks after treatment. Therefore, the total effective rate of less than or equal to 2 weeks was reported in 8 studies [30–33, 35–37, 39] and that of more than 2 weeks was reported in 4 studies [35, 37, 38, 40]. The total effective rate (less than or equal to two weeks) had statistical significance (OR = 2.84; 95% CI [1.91, 4.23]; *P* < 0.00001; *I*^2^ = 19%) with low heterogeneity (Figure 4). The total effective rate (more than two weeks) had statistical significance (OR = 1.91; 95% CI [1.11, 3.27]; *P*=0.002; *I*^2^ = 0%) with low heterogeneity (Figure 5). The result showed that thunder-fire moxibustion had a better effect compared with the control group.

### 4.2. Traditional Chinese Medicine Syndrome Score

**Figure 6:**
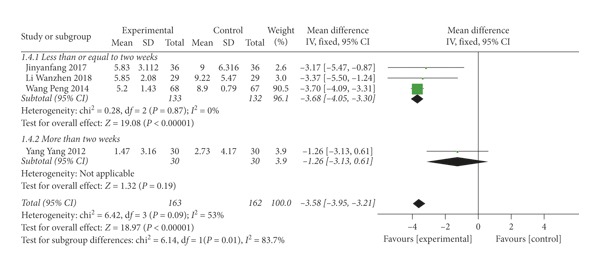
Forest plots of traditional Chinese medicine syndrome score.

The score of TCM syndrome was assessed in 4 trials [31, 32, 36, 38], which was scored according to the severity of symptoms. The result showed that SMD = −3.58 (*P* < 0.00001), indicating that thunder-fire moxibustion was more effective than the control group; however, there was substantial heterogeneity (*I*^2^ = 53%) (Figure 6). With the purpose of finding the source of heterogeneity, sensitivity analysis was conducted. The heterogeneity dropped to 0% after removing Yang Yang research. After comparison, we speculated that the heterogeneity may be caused by the single treatment time. The single treatment time of this study was 10 min, while the single treatment time of remaining studies was more than or equal to 20 min. We performed subgroup analysis according to the treatment days. We divided the trials into two groups, one is less than or equal to two weeks, the other is longer than two weeks. Three studies [31, 32, 36] in the subgroup of “less than or equal to two weeks” revealed a statistical significance (SMD = −3.68; 95% CI [−4.05, −3.30]; *P* < 0.00001; *I*^2^ = 0%).

### 4.3. Pain Score

**Figure 7:**
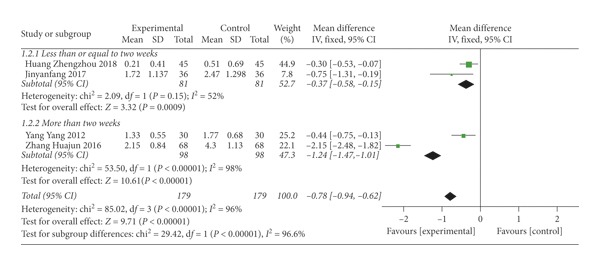
Forest plots of pain score.

Four trials [30, 31, 38, 40] reported pain sore, two [38, 40] of them used the NRS and the other two [30, 31] used the VAS. The higher the score was, the more serious the condition was. The result showed that SMD = −0.78 (*P* < 0.00001), indicating that thunder-fire moxibustion was more effective than the control group; however, there was substantial heterogeneity (*I*^2^ = 96%) (Figure 7). We performed sensitivity analysis to find sources of heterogeneity. The heterogeneity dropped to 10% after removing Zhang huajun research. We conjectured that herb was used in both groups in this research, while other researches did not use the herb. Two trials [38, 40] assigned to the group less than or equal to two weeks, which were the same trials evaluated by the NRS, revealed a statistical significance (SMD = −0.37; 95% CI [−0.58, −0.15]; *P*=0.0009; *I*^2^ = 52%). Two more trials [30, 31] assigned to the group more than two weeks ago, which were the same trials evaluated by the VAS, revealed no statistical significance (SMD = −1.24; 95% CI [−1.47, −1.01]; *P* < 0.00001; *I*^2^ = 98%).

### 4.4. Satisfaction Score

**Figure 8:**
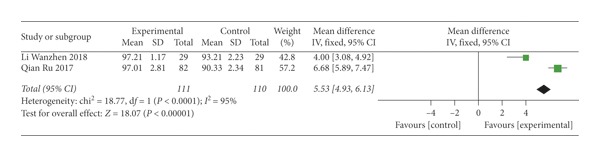
Forest plots of satisfaction score.

Two studies [32, 33] assessed satisfaction score of patients. The higher the score was, the better the treatment effect was. The result showed that SMD = 5.35 (*P* < 0.0001), indicating that thunder-fire moxibustion was more effective than the control group; however, there was substantial heterogeneity (*I*^2^ = 95%) (Figure 8).

### 4.5. Score of the Symptoms and Functional Rehabilitation of Cervical Vertigo

**Figure 9:**
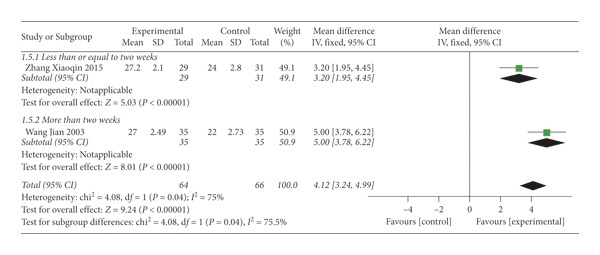
Forest plots of score of the symptoms and functional rehabilitation of cervical vertigo.

Two studies [34, 41] assessed the score of the symptoms and functional rehabilitation of cervical vertigo. The higher the score was, the better the treatment effect was. The result showed that SMD = 4.12 (*P* < 0.00001), indicating that thunder-fire moxibustion was more effective than the control group; however, there was substantial heterogeneity (*I*^2^ = 75%) (Figure 9).

### 4.6. Adverse Events

Two trials [30, 36] reported adverse reactions. Huang [30] reported one case of burning pain but no scalds and two cases of mild cough which relieved after proper ventilation. Wang [36] reported no discomfort of the gastrointestinal tract, peripheral blood vessels, and tissues in the experiment group. The routine examination of blood, urine, stool, and liver and kidney functions was normal.

### 4.7. Thunder-Fire Moxibustion Performed for CS

Acupoints, treatment times, and courses used in various studies were not identical. Thunder-fire moxibustion was performed every day in all included studies. The two studies measured in the second and third weeks [35, 37]. Except 3 studies [30, 34, 40] that did not report the single treatment time, 3 studies [31, 36, 41] had a 30 min of treatment duration, 2 studies [32, 33] had a 20–30 min of treatment duration, 2 studies [35, 37] of 20 min, and 2 studies [38, 39] of 10 min or more than 10 min (Table 2).

We had analyzed the use of acupoints. There were 16 acupoints used in 12 trials. The two studies [30, 40] have the same acupoint selection scheme, and two more studies [32, 33] had another same acupoint selection scheme, while the remaining studies had different acupoint selection schemes. Go14 (10 studies [30–33, 35–37, 39–41], 83.3%) had the highest frequency of use, followed by GB20 (8 studies [31, 34, 36, 41], 66.7%), EX-B2 (5 studies [30, 35, 37, 38, 41], 41.7%), GB21 (5 studies [30–33, 40], 41.7%), and ashi point (4 studies [31, 35, 37, 38], 33.3%). The other acupoints were used three times or less (Table 3).

### 4.8. Publication Bias

**Figure 10:**
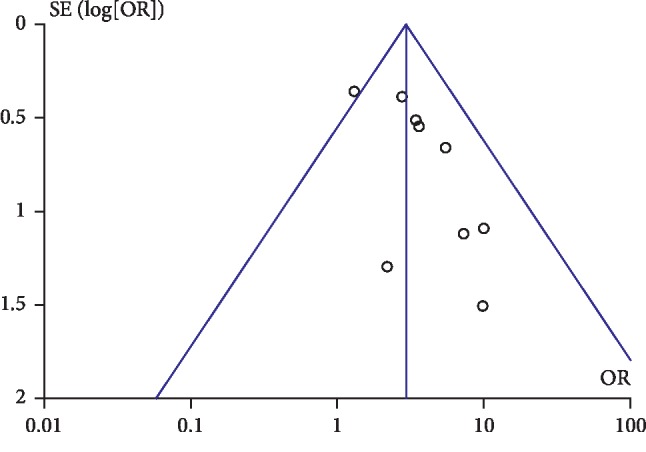
Funnel plot of the publication bias.

We used RevMan to perform publication bias only on the total effective rate, which included 10 studies. These 10 studies were performed in China, and the sample sizes were small. The results showed that the funnel plot was asymmetrical, illustrating that there may be publication bias in the efficacy of thunder-fire moxibustion in the treatment of CS (Figure 10). For a long time, the asymmetry of the funnel plot has been regarded as publication bias, but the funnel plot should be regarded as a general means to show the effect of small-scale research. In addition to publication bias, there are other factors that may lead to small-study effect, such as the exaggeration of results in small researches due to the low quality of methodology, real heterogeneity, and accidental factors.

### 4.9. Level of Evidence

We assessed the evidence quality with the aid of GRADE. The quality of evidence for all comparisons was very low, limiting our recommendation of research findings. There were five factors that can reduce the level of evidence: risk of bias, imprecision, indirectness, inconsistency, and publication bias. The risk of bias was assessed to be high. The inconsistency was low in all researches. All studies compared treatment outcomes straight, so indirectness did not downgrade. Several comparisons displayed high heterogeneity to downgrade the evidence level. Small sample sizes in most studies led to imprecision and publication bias. Of the three escalation factors, none of the studies achieved. There upgrading factors, which were dose-response gradient, large effect, and plausible confounding, were not achieved in all studies (Table 4). Due to the above 8 factors, the quality of evidence in all comparisons was very low.

## 5. Discussion

We want to appraise the curative effect and safety of thunder-fire moxibustion on CS. We included 12 RCTs for meta-analysis after searching and screening the major domestic and foreign database by evidence-based medicine. The result showed that thunder-fire moxibustion was more effective than the control group. Thunder-fire moxibustion can improve the total effective rate of treatment and relieve pain, numbness, and other symptoms of cervical spondylosis. We recommend the effect of thunder-fire moxibustion in CS finitely because of the very low level of evidence. Traditional Chinese medicine syndrome score, pain score, satisfaction score, and score of the symptoms and function rehabilitation of cervical vertigo were statistically significant with substantial heterogeneity. Although subgroup analysis was conducted, heterogeneity still existed in these comparisons. The variety of acupoint selection scheme and therapeutic manipulation may have caused the unresolved heterogeneity. The evidence quality was very low. Due to the low methodology quality of the included literature and the small sample size and the fact that they were all carried out in one country, the conclusion of the composite outcome was not very reliable.

Only one trial reported adverse reactions such as burning pain and mild cough. Routine examination of blood, urine, stool, and liver and kidney functions were normal. We can not draw a conclusion on the safety of thunder-fire moxibustion due to the insufficient number of studies included.

In this research, we have seen that thunder-fire moxibustion alone or associated with other therapies had a similar curative effect. Due to the following limitations, we were incapable of reaching an exact finding regarding the efficacy of thunder-fire moxibustion. The methodological quality of inclusive researches was low. Despite the comprehensive database search, only studies performed in China were included, leading to publication bias. Only few studies had reported the generation of random sequences. The sample size of most researches was small. There was no multicentre study, and the outcome indicators were subjective. All studies had not performed allocation concealment, blinding of participants, subjects, and outcome measurers, which would lead to high risks of selection bias and performance bias, along with detection bias. These factors exaggerate the efficacy of thunder-fire moxibustion in CS [42, 43]. Only one research reported the results of one-month follow-up after treatment. One research reported that one case of thermalgia but not scalds and two cases of slight cough.

The potential mechanism of thunder-fire moxibustion for CS is not yet distinct, but it does have a positive therapeutic effect in CS. Chen [44] found that thunder-fire moxibustion effectively reduced the contents of TNF-*α* and IL-1*β* in serum in the rat model of knee osteoarthritis, thereby inhibiting inflammatory response and alleviating symptoms. Thunder-fire moxibustion is widely used in China in treating any disease, including pain caused by a variety of reasons [45–47]. In general, the normal thunder-fire moxibustion operation will not cause invasive injury to patients. Noninvasive treatment can increase participant compliance. Compliance to a certain extent affects the successful implementation of the experimental scheme, the clinical research quality, as well as the dependability of test results [48, 49]. The mechanism, efficacy, and safety of thunder-fire moxibustion deserve intensive study.

There are some limitations and shortcomings in the study. Only few trials of low methodological quality were found, and all were performed in China. The follow-up data were deficient to assess the long-term efficacy. Owing to the diversity of interventions, there were a fewer trials comparing each intervention, and sample sizes were also reduced for each comparison. Meanwhile, this research has not analyzed the different categories of acupoint selection schemes, moxibustion method, or duration, which may influence the findings. Less reporting of adverse reactions can also lead to bias. It was difficult to draw an exact conclusion because of the lack of trials.

Researchers need to consider the following factors for future research. Randomized controlled trials designed rigorously with a large sample size should be performed. The objective index and follow-up information of thunder-fire moxibustion are still insufficient, so we advise that long-term clinical trials with objective index should be performed in future to access the efficacy of thunder-fire moxibustion. Strict protocol is required before performing research, and researchers need to report on the basis of systematic reporting guidelines, for example, Consolidated Standards of Reporting Trials (CONSORT) and Extension for Herbal Interventions 2006 [50]. At the same time, researchers should use CONSORT in Harms Data Recommendations to detect and report adverse reactions in future studies. As the mechanism of thunder-fire moxibustion in relieving CS has not yet been established, researchers should pay attention to this aspect in future research.

## 6. Conclusion

Based on the existing evidence, the curative effect of thunder-fire moxibustion in CS is weak. Thunder-fire moxibustion has a fewer side effects and is relatively safe. In addition, thunder-fire moxibustion is noninvasive and comfortable and can improve patient compliance. The methodological quality of the relevant researches is low due to small sample size and rough experimental design. Therefore, future researchers should perform large-sample, well-designed, long-term RCTs to assess the efficacy and safety and the mechanism of thunder-fire moxibustion.

## Figures and Tables

**Table 1:** Search strategy.

Number	Search terms
01	Cervical spondylosis
02	Spondylosis, cervical
03	Spondylosis deformans
04	Or 01–03
05	Thunder-fire moxibustion
06	Leihuo moxibustion
07	Or 05–06
08	Randomized controlled trial
09	Randomized trial
10	Randomly
11	Trial
12	Or 08–11
13	04 and 07 and 12

**Table 2:** Characteristics of the included studies.

First author (year)	Number of participants, E:C	Completion number, E:C	Mean age (range) (year)	Experiment	Control	Duration	Outcomes	Moxibustion acupoint	Moxibustion method
Huang 2018 [30]	45 : 45	45 : 45	E: 39.32 ± 10.33 (20–61) C: 39.49 ± 11.68 (20–61)	*a*	*b*	5 d, NR	①, ③	EX-B2, GB20, GB21, Go14	Manipulation

Jin Zhu 2017 [31]	36 : 36	36 : 36	E: 48.05 ± 11.22 C: 49.28 ± 8.70	*a* + *c*	*c*	10 d, 30 min/d	①, ②, ③	GB21, Go14, ashi point	Array

Li 2018 [32]	29 : 29	29 : 29	E: 49.26 ± 5.37 (26–78) C: 50.18 ± 4.29 (28–76)	*a* + *d*	*d*	10 d, 20–30 min/d	①, ②, ④	LI4, LI11, jianshu, GB20, GB21, Go14, Go16	Manipulation

Qian 2017 [33]	82 : 81	82 : 81	E: (25–83) C: (25–83)	*a* + *d*	*d*	14 d, 20–30 min/d	①, ④	LI4, LI11, jianshu, GB20, GB21, Go14, Go16	Manipulation

Wang 2003 [34]	35 : 35	35 : 35	E: 48 ± 7.91 C: 47 ± 8.81	*a* + *e*	*c* + *e*	20 d, once a day	⑤	St9, St13, St36, St41	Array

Wang 2012 [35]	91 : 91	91 : 91	E: (30–70) C: (30–70)	*a* + *f*	*c* + *g*	21 d, 20 min/d	①	EX-B2, GB20, Go14, ashi point	Manipulation

Wang 2014 [36]	70 : 70	68 : 67	E: 54.95 ± 7.15 C: 52.50 ± 6.10	*a* + *g*	*g*	10 d, 30 min/d	①, ②	BL10, BI11, Go14, Go15, Go16	Manipulation

Xiao 2011 [37]	126 : 126	126 : 126	E: (30–70) C: (30–70)	*a* + *f*	*c* + *g*	21 d, 20 min/d	①	EX-B2, GB20, Go14, ashi point	Manipulation

Yang 2012 [38]	30 : 30	30 : 30	E: 45.69 ± 7.58 (18–65) C: 46.73 ± 8.03 (18–65)	*a* + *c*	*c*	20 d, 10 min/d	①, ②, ③	EX-B2, GB20, ashi point	Manipulation

Zeng 2010 [39]	13 : 13	13 : 13	NR	*a* +*d*	*g*	10 d, ≥10 min/d	①	GB20, Go14	Manipulation

Zhang 2016 [40]	68 : 68	68 : 68	E: 43.2 ± 4.0 (25–70) C: 43.2 ± 4.3 (25–69)	*a* + *c* + *g*	*c* + *g*	20 d, NR	①, ③	EX-B2, GB20, GB21, Go14	Array

Zhang 2015 [41]	29 : 31	29 : 31	E: 56.5 ± 12.7 (45.8–69) C: 6.5 ± 12.78 (45.8–69)	*a* + *g*	*g*	10 d, 30 min/d	⑤	Go14	Array

E, experimental group; C, control group; NR, not reported; *a,* thunder-fire moxibustion; *b,* moxibustion; *c,* acupuncture; *d,* routine nursing; *e,* physiotherapy; *f,* needle knife; *g,* medicine; ① total effective rate; ② traditional Chinese medicine syndrome score; ③ pain score; ④ satisfaction score; ⑤ score of the symptoms and functional rehabilitation of cervical vertigo.

**Table 3:** The most frequently used acupoint.

Order	Acupoints	Frequency (%, *N* = 12)
1	Go14	10 (83.3%)
2	GB20	8 (66.7%)
3	EX-B2/GB21	5 (41.7%)
4	Ashi point	4 (33.3%)
5	Go16	3 (25.0%)
6	LI4, LI11, jianshu	2 (16.7%)
7	St36, St41, St9, St13, BL10, BI11, Go15	1 (8.33%)

**Table 4:** Level of evidence.

Variable	Effect (OR/MD)	95% CI	*P*	*I* ^2^(%)	*P* (*X*^2^test)	Statistical method	Studies (*N*)	Sample size (*N*)	Level of evidence
Total effective rate (less than or equal to two weeks)	2.84	1.91, 4.23	<0.00001	19	0.28	Fixed effect models	8	978	Very low

Total effective rate (more than two weeks)	1.91	1.11, 3.27	0.02	0	0.54	Fixed effect models	4	630	

Traditional Chinese medicine syndrome score	−3.58	−3.95, −3.21	<0.00001	53	0.09	Fixed effect models	4	325	Very low

Less than or equal to two weeks	−3.68	−4.05, −3.30	<0.00001	0	0.87	Fixed effect models	3	265	Very low

Pain score	−0.78	−0.94, −0.62	<0.00001	96	<0.00001	Fixed effect models	4	358	Very low

Less than or equal to two weeks	−0.37	−0.58, −0.15	0.0009	52	0.15	Fixed effect models	2	162	Very low

Satisfaction score	5.53	4.93, 6.13	<0.00001	95	<0.0001	Fixed effect models	2	221	Very low

Score of the symptoms and function rehabilitation of cervical vertigo	4.12	2.34, 4.99	<0.00001	75	0.04	Fixed effect models	2	70	Very low

## Abbreviations

CS: cervical spondylosis
PRISMA: preferred reporting items for systematic reviews and meta-analyses
RCTs: randomized controlled trials
NRS: numeric rating scale
VAS: visual analog scale
WFDP: Wanfang digital periodicals database
CNKI: China national knowledge infrastructure
CBM: Chinese biomedical literature database
TCM: traditional Chinese medicine.

## Supplementary Materials

Supplementary Materials PRISMA 2009 Checklist.
